# Parents’ Perceptions of a Serious Game for Educating Families on Prescription Opioid Safety: Qualitative Pilot Study of MedSMARxT: Adventures in PharmaCity

**DOI:** 10.2196/49382

**Published:** 2023-09-12

**Authors:** Olufunmilola Abraham, Grace Ann Nixon, Laura Louise Seitz

**Affiliations:** 1 Social and Administrative Sciences Division School of Pharmacy University of Wisconsin-Madison Madison, WI United States

**Keywords:** opioids, opioid, parents, adolescents, medication safety, family health, serious games, parent, adolescent, youths, gaming, game, games, teenager, teenagers, acceptance, perception, perceptions, patient education, pharmacy, pharmaceutic, pharmaceutics, pharmaceutical, drug, safety

## Abstract

**Background:**

Opioid misuse is a pervasive, worsening problem that affects the health of people throughout the United States, including adolescents. There are few adolescent-focused interventions designed to educate them about opioid medication safety. The MedSMARxT: Adventures in PharmaCity, is a serious educational video game that teaches parents and their youths about safe opioid practices.

**Objective:**

This study aimed to elucidate parent’s perceptions of MedSMARxT: Adventures in PharmaCity and its potential use by parents and their adolescents.

**Methods:**

Parents of adolescents aged 12 to 18 years who live in the United States were recruited from April to October 2021 via Qualtrics research panels, social media, email listserves, and snowball sampling. The study participants played MedSMARxT: Adventures in PharmaCity for 30 minutes and then participated in a 30-minute postgame interview via WebEx (Cisco). Questions were developed and piloted to examine adults’ perceptions of the game. Participants were asked three sets of open-ended questions: (1) questions about the game and elements of the game, (2) what they learned from the game, and (3) questions about their experience with games. Audio recordings were transcribed verbatim. Interview transcripts were coded using content and thematic analysis by study team members to identify major themes and subthemes from the data.

**Results:**

Parent participants (N=67) played MedSMARxT: Adventures in PharmaCity and completed a postgame interview. Analysis extrapolated four primary themes from the data: (1) participant gaming experience, (2) perception of game features, (3) educational purpose of the game, and (4) future use of the game. Most participants (n=56, 84%), had at least some experience with video games. More than half of the participants (n=35, 52%) participants, had positive reactions to the game characters and scenes depicted in MedSMARxT: Adventures in PharmaCity and stated they were realistic for adolescents. Most participants (n=39, 58%), would recommend the game to others. Significant difficulties with gameplay navigation were reported by 38 (57%) participants, as well as a slow game pace. All participants were able to accurately identify the overarching goal of the game: opioid or medication safety. The game reinforced existing knowledge for participants, though many (n=15, 22%), reported a new awareness of the need to store opioid medications in a locked area and the availability of medication disposal drop boxes at pharmacies. Participants stated that they would recommend the game for future use by families and youths in various health care and non–health care settings.

**Conclusions:**

The use of a tailored serious game is a novel, engaging tool to educate adolescents on opioid safety. MedSMARxT: Adventures in PharmaCity can be used as a tool for parents and adolescents to facilitate meaningful dialogue about safe and appropriate opioid use.

## Introduction

### Background

The opioid crisis has palpable impacts throughout the United States across all demographics. Seldom can one go a significant amount of time without hearing about the epidemic’s dire impacts on health and well-being. Death rates from opioid misuse have nearly tripled since 1999, and opioids were involved in an estimated 80,816 deaths in the United States in 2021 alone [1,2]. Curbing opioid misuse has been a long-standing focus of policy and governmental efforts, with the White House declaring the opioid epidemic a public health emergency in 2017 [3].

Youths are just as vulnerable to the growing opioid crisis as adults. In a survey collected by the Centers for Disease Control and Prevention in 2019, approximately 14% of high school students reported misuse of prescription opioids during their lifetime [4]. Opioid misuse encompasses taking opioids outside of their prescribed regimen, such as taking more than prescribed, using them in the absence of pain, or taking them after expiration. Among adolescents, misuse often begins at home, where leftover opioid medications enable misuse [5,6]. Adolescents tend to model the behaviors of their parents regarding their opioid prescription usage [7]. Research shows that the proportion of adolescents and young adults with opioid-related health problems has increased over time despite a decrease in the total number of adolescent and young adult personal opioid prescriptions during the same period [8]. Out of all the adolescents and young adults who experienced an opioid-related problem, 40% had received a personal prescription for opioids in the past year while 48% had a family member prescribed an opioid in the past year. Even adolescents who are prescribed opioids and take them appropriately are 33% more likely to develop problems such as opioid dependence or lifelong patterns of misuse [1].

However, research has shown that educating parents can improve their behavioral intentions and reduce the likelihood that they will store unused opioid medications in the home [9-11]. Alongside rising rates of adolescent opioid misuse, youths have reported misconceptions about safe opioid use practices, including inaccurate identification of medications that are opioids, improper storage and disposal techniques, and inappropriate sharing of prescribed opioids with others [12]. Due to the vulnerability of adolescents and their limited knowledge about opioid medication safety, it is essential to create tailored, educational interventions that teach youths and families about the importance of safe prescription opioid use.

Currently, there is a lack of interventions tailored to adolescent education on opioid medication safety [13-16]. Previous literature suggests that parents and youths are receptive to game- and web-based opioid misuse prevention programs, citing a belief that gamified educational methods would improve adolescent engagement [17,18]. These studies have also shown that serious educational games may enhance the retention of information, accelerate learning, and increase accessibility to educational material. Other health-focused games have shown effectiveness in improving key behavioral intentions and improving education for youths [18-21]. Novel approaches to engaging youths can be effective and are paramount in combating the opioid crisis [22,23]. One such approach is the use of a serious game. Serious games are video games designed to impart knowledge or improve social-behavioral outcomes for players wherein the primary objective is not entertainment [24].

### MedSMARxT: Adventures in PharmaCity

#### Overview

MedSMARxT: Adventures in PharmaCity is a theory-driven, adolescent-focused serious game developed to educate adolescents and their families on how to safely manage complex, real-life situations involving prescription opioid medications [25,26]. By playing the game, youths and their family members (such as parents) are able to identify best practices for the safe and responsible management of opioid medications with a focus on appropriate disposal, proper storage, and preventing medication sharing. The game scenarios were created with the interdisciplinary study team comprised of game designers and developers, health services researchers, pharmacists, an adolescent health physician, and an addiction medicine physician. The scenarios were further assessed by adolescents and young adults who overall found the scenarios to be realistic and relevant. The game content, game design, and scenarios were informed by several extensive studies previously conducted by the study team to understand opioid safety knowledge games of adolescents and their preferences for game-based education [9,14-16,25,26]. Findings from these studies showed that adolescents significantly lacked information on safe opioid storage and disposal; hence, a critical knowledge gap that needed to be addressed via game-based learning. Parent involvement in medication-related behaviors (such as medication storage and disposal) remains an important protective factor against adolescent prescription medication misuse [27]. Therefore, the inclusion of parents in the feedback and development was key to creating an intervention that would facilitate adolescent education and promote family medication safety.

The game’s format was a responsive narrative wherein players make choices that affect the game’s outcomes, such as choosing their own adventure. Players play as an anthropomorphized sheep, Shan, who recently broke their arm. Players must use critical thinking and decision-making to select the correct (safest) choices regarding opioid use through 5 levels simulating real life. If players make a decision that is unsafe, they are presented with an opportunity to replay the level. The game is played using an internet-enabled computer via a web browser.

#### Level 1

**Figure 1:**
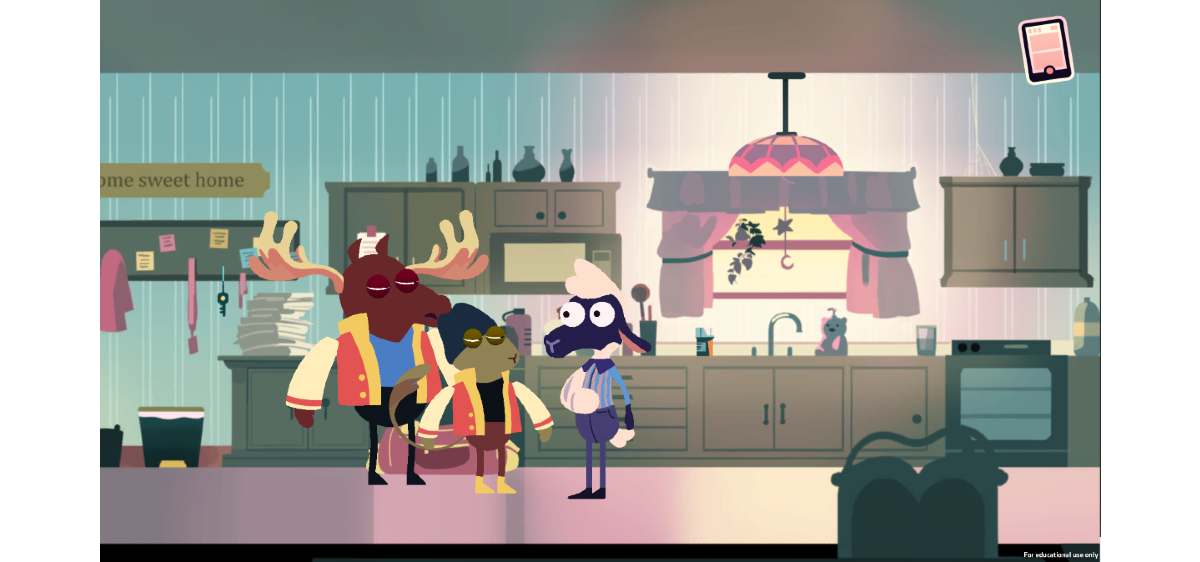
Game level 1.

A Quiet Sunday Afternoon focuses on teaching the player about the safe storage of opioids and requires the player to appropriately store opioids in a locked cabinet before Shan’s friends gain access to the medications and overdose (Figure 1).

#### Level 2

**Figure 2:**
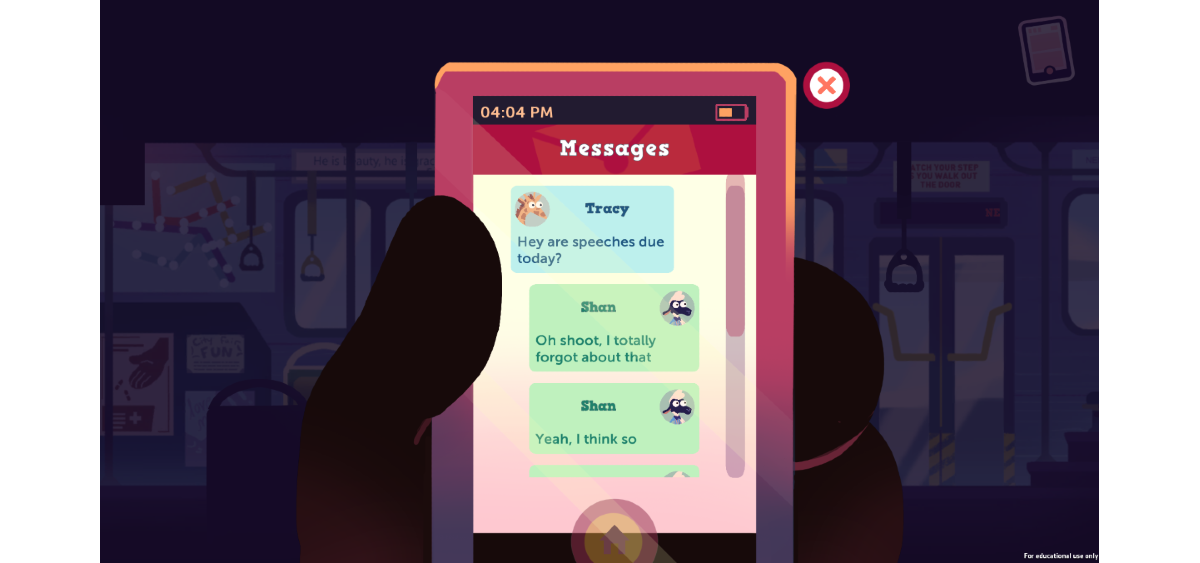
Game level 2.

Monday Morning Bus Ride, in this scene, the player is in pain and forgets there was an important assignment due that day (Figure 2).

#### Level 3

**Figure 3:**
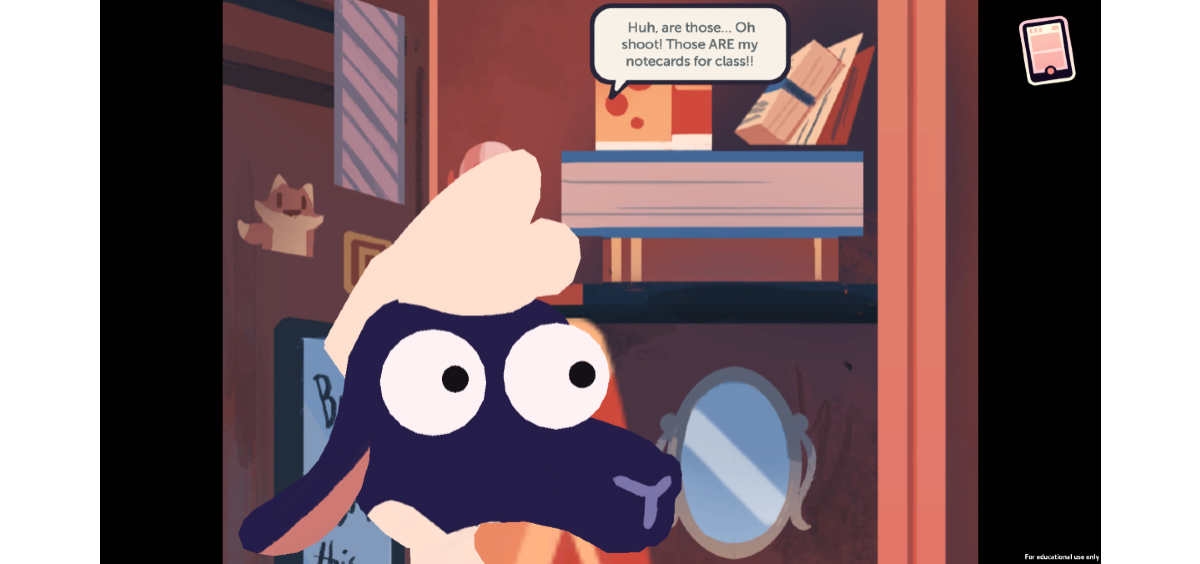
Game level 3.

A Persuasive Speech at School teaches the player not to take others’ pain medications through peer pressure from a friend and discusses the negative consequences of taking someone else’s prescription medication (Figure 3).

#### Level 4

**Figure 4:**
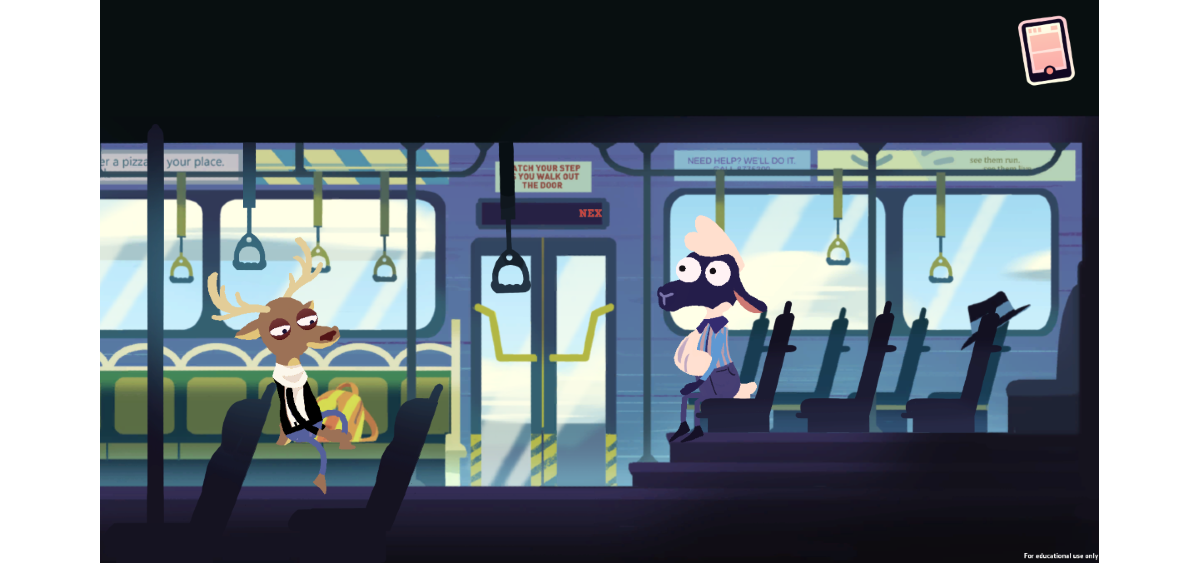
Game level 4.

Bus Ride Home teaches the player not to share their medication with others and reinforces the risks of sharing medications. This game level also introduces the use of Narcan through an encounter with a stranger on the bus who acts as if they are in pain and needs relief (Figure 4).

#### Level 5

**Figure 5:**
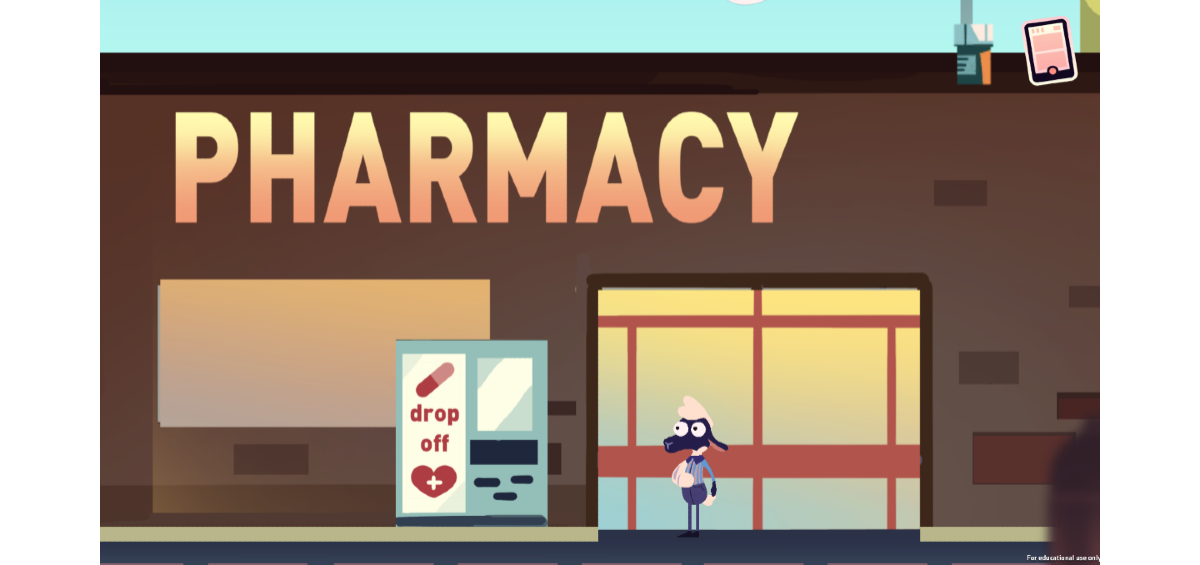
Game level 5.

Last Minute Chore shows the player the correct approach to dispose of opioid medication and that medication disposal drop boxes are available at local pharmacies (Figure 5).

### Study Objectives

The purpose of this study was to examine parents’ perceptions of the MedSMARxT: Adventures in PharmaCity game. Specifically, the study assesses parents' perceptions of the game features, what participants learned from the game, and their gaming experience.

## Methods

### Ethics Approval

This study was approved by the University of Wisconsin-Madison’s institutional review board (2020-1638) on January 5, 2021.

### Sampling and Recruitment

From April to October 2021, a national sample of parents was recruited via Qualtrics research panels, social media, email listserves, and snowball sampling. The study team partnered with Qualtrics to identify and recruit from preexisting research panels. Social media sites included Facebook (Meta Platforms), Instagram (Meta Platforms), Reddit, and Twitter. Listserves came from the University’s mass email system and the study team’s own list of youths and parents. Eligible participants were parents of adolescents aged 12 to 18 years who live in the United States, could read, speak, and understand English, and had access to a computer with a working webcam. After screening for eligibility, parents were asked to provide consent and their contact information for scheduling purposes. Parents were then contacted to partake in the study and screened for eligibility by the research staff.

### Data Collection

Study sessions were held via WebEx. Parents were told that the study was being conducted by a team of researchers interested in adolescent opioid misuse prevention and that they were looking for data to help refine their game. Parents were asked to play the MedSMARxT: Adventures in PharmaCity game for 30 minutes (or until they completed all 5 levels, whichever happened first), and answer follow-up interview questions. Data were collected through 30 minutes of gameplay and a 30-minute semistructured interview. Only participants and a member of the research staff were present during the data collection. The research team created interview questions to examine parent players’ feedback on the game. Participants were asked three sets of open-ended questions: (1) about the game and elements of the game, (2) what they learned from the game, and (3) about their experience with games. Interview questions were developed for the purpose of understanding parents’ perceptions of the game, confirming the salience of educational content, and characterizing the gaming experience of parents of adolescents. The study team used interview questions that had previously been pilot-tested and proved to be effective for examining adults’ use of the game. The interview ended with, “Is there anything else you’d like to add?” to allow the participant to share any other thoughts or details that were not previously captured. Parents were compensated US $30 via an Amazon gift card for their participation in the study. Interviews were professionally transcribed verbatim using audio recordings collected through WebEx. The interview guide is shown in Multimedia Appendix 1.

### Data Analysis

Two research team members (GAN and LLS) independently analyzed each interview transcript using NVivo (Lumivero) [28]. The main themes from participant responses were coded using content analysis followed by a thematic approach [29]. The coding members of the study team first familiarized themselves with the data by reading through each transcript in detail and then independently extrapolated codes. The research team members met regularly to discuss coding differences and develop a master codebook. The codes were then exported to Excel (Microsoft Corp) for further thematic analysis using an inductive and deductive approach to identify salient themes [30]. The primary investigator (OA) met with the coders to address any coding discrepancies and prevalent themes identified. Each coder identified themes and subthemes from the data based on code prevalence, and then the research team met to compare and finalize the results reported below.

## Results

### Participant Demographics

Feedback was elicited from 67 parent participants who played MedSMARxT: Adventures in PharmaCity. Participant characteristics are described in Table 1. Notably, 92.5% (n=62) of them identified as female, 77.6% (n=52) of them identified as White, and the mean age was 46.03 (SD 5.75) years.

As depicted in Textbox 1 below, four major themes were identified: (1) participant gaming experience, (2) perception of game features, (3) educational purpose of the game, and (4) future use of the game. A detailed description of the themes and prevalent subthemes that emerged from the data analyses is described below.

**Table 1:** Participant demographics (N=67).

Characteristics		Values
**Level of education, n (%)**		
	High school	3 (5)
	Associates or trade school	8 (12)
	Bachelor's degree	26 (39)
	Master's or PhD	30 (45)
Age (years), mean (SD)		46.03 (6)
**Sex^a^, n (%)**		
	Female	62 (93)
	Male	5 (8)
**Race or ethnicity^b^, n (%)**		
	White	52 (78)
	Black or African American	7 (10)
	Hispanic or Latinx	3 (5)
	Asian	2 (3)
	More than one selected	3 (5)
	Other please specify	0 (0)
**Income (US $), n (%)**		
	Under $25,000	2 (3)
	$25,001-$50,000	8 (12)
	$50,001-$100,000	23 (34)
	$100,001-$250,000	32 (48)
	$250,001-$500,000	2 (3)
**Employment status, n (%)**		
	Part-time, unemployed, retired, and other	34 (51)
	Full-time	33 (49)
**Regions^c^, n (%)**		
	Atlantic	16 (24)
	Central	28 (42)
	Southern	16 (24)
	Western Pacific	7 (10)

^a^Three options were presented to the participants to select for their sex assigned at birth: “male,” “female,” and “other: please specify.”

^b^If participants only selected 1 category, that was their defined race; all other combinations of selections were defined as “More than once selected.”

^c^Regions are generated from participant zip codes according to the United States Postal Service data.

**Summary of themes and subthemes.**

**Participant gaming experience**

- Experience with board and card games
- Experience with video games
- Experience with educational games

**Perception of game features**

- Levels
- Characters
- Graphics
- Navigation
- Pace

**Educational purpose of the game**

- Perceived main idea of the game
- Reading level
- Target audience
- Participant learning experiences

**Future use of game**

- Suggested improvements
- Applications

### Theme 1: Participant Gaming Experience

#### Overview

Parents had a wide range of experience with games. Overall, participants preferred games that required strategy and discrete tasks to accomplish. Study participants enjoyed games that foster family or social interaction. Most parents reported at least some experience with video games and favorable impressions of educational games.

#### Experience With Board and Card Games

Many participants (n=32, 48%), reported playing board games, and overall, participants listed 70 different board games that they had played in the past. Social interaction with family and friends was a frequent motivator for participants to play board games. A smaller number of participants (n=13, 19%), discussed playing card games.

I play them with my family so it's something fun to do with my family or with my friends. […] So, it's more of a social thing for me, it's not a sit on my phone or computer and do something by myself.

Parent 152

I'm not a competitive person, but I like having something to do while you're sitting around and talking with someone else.

Parent 184

#### Experience With Video Games

Most participants (n=56, 84%), stated they had at least some video gaming experience. A few participants (n=3, 4%), considered themselves frequent players, and nearly half of the participants (n=30, 45%), considered themselves infrequent players. Of some participants (n=11, 16%), reported minimal to no experience with video games. Participants had used a variety of gaming platforms and systems, but mobile gaming was most frequently reported. Participants listed a total of 132 unique video games played. Common types of video games played include word, puzzle, and matching games.

Motivations for playing video games included a desire to pass time when relaxing or bored, to have a mental challenge, and to socialize with others, especially participants’ own children. Lack of time, preferences for other forms of entertainment, and avoidance of violent themes were the 3 most frequent reasons for not playing video games. Some participants (n=18, 27%), stated nothing could be changed about video games to entice them to play more. Furthermore, a few participants (n=5, 7%), reported easier navigation would increase appeal and 5 (7%) participants mentioned that including goals would motivate them to play.

Yes, I have experience playing. I mean, I'm not sitting there every day playing, but I do play with the kids here and there. I like to mostly use my phone for games, but we do have consoles like the Xbox 1.

Parent 194

I'm really busy and I like to do other things with my time. I spend a lot of time on the computer at work and so when I come home, it's like, the last thing I want to do is spend more time on the computer.

Parent 144

#### Experience With Educational Games

Most participants (n=44, 66%), reported previous experience playing an educational game. Some of these participants (n=11, 16%), reported playing an educational game with their child in the past, and other parents (n=11, 16%), stated that educational games were a part of their career. Participants listed 36 unique educational games that they had previously played, which commonly included math games, crossword puzzles, early learning games, and trivia games.

Most parents (n=43, 64%), had a positive perception of educational games. Study participants perceived game-based learning as an appealing modality that is more engaging than traditional learning methods. Features such as the ability to learn from mistakes within the game and entertainment features were important to parents. A few participants (n=7, 10%), stated that educational games would be more beneficial for youths than adults.

I think it's really good. I mean, obviously, depending on what you're being educated on. But I think is a great way to learn and also kind of keep your interest as opposed to just reading it on the Internet or reading a book about it. It’s a way to, a different way to learn to kind of keep it a little spicy, I guess.

Parent 163

I feel like your kids and adults enjoy playing them and have fun with them without realizing that it's a learning process because sometimes if you're like, “hey, we're going to teach you something,” or “this is going to be educational,” people automatically go to like, “this is going to be really boring.” Um, so the good educational games kind of distract you from the fact that you're learning something by making it mostly fun. For younger kids, that's especially good.

Parent 161

### Theme 2: Perception of Game Features

#### Levels

The most frequent positive descriptors of game levels included “cute” and “engaging.” Participants appreciated the variety of game levels, which they reported represented real life. Parents appreciated the responsive narrative format which allowed the player to choose from multiple available actions, learn the consequences of wrong (unsafe) actions, and replay scenes to discover the appropriate actions.

A total of 35 (52%) participants reported that game levels were realistic. Some participants (n=10, 15%), specifically stated that the levels were applicable to the lives of adolescents. Only 3 (4%) participants reported that game scenes were not realistic due to the severity of the consequences portrayed. Some parents expressed uncertainty about whether adolescents encountered opioid misuse frequently. A small number of 3 (4%) participants noted that in the game a child’s character performs actions they would not have their own child perform, such as medication disposal.

In level 1, some participants reported uncertainty around the correct actions to take, though many benefited from the educational content of this level. Level 3 was well-received by participants due to its depiction of an opioid misuse scenario with peer pressure which they felt adolescents may encounter. Level 4 was sometimes viewed as being unlikely, as participants felt that a stranger would not ask for pain medication from another person.

We don't necessarily know what kids are thinking. […] The conversation between Stan and Tracy was very interesting because you would never think […] that another teenager would offer their friend opioids, trying to help them out, nothing nefarious about it. But it's very dangerous. I mean, I could see how it could happen.

Parent 199

I think there were good choices, especially with the things at home and then the potential at school with somebody else having other medications. I liked that they were more of like, situations that kids might find themselves in, not trying to so, like, not actually having that seeking behavior, or, like, trying to purchase them from somewhere, but just happening, especially like, with the friend offering.

Parent 172

I think it's kind of realistic, but I haven’t experienced or heard like from my kids or my family and my friends, I never heard this sharing the high-dose painkiller opioid in some, this kind of things. So, I will be neutral. I don't say it’s realistic or not realistic.

Parent 188

#### Characters

Parents responded positively to the game’s characters. Parents’ positive impressions of the characters were mentioned 166 times, whereas negative impressions of characters were noted only 38 times. Characters were described as cute, realistic, and sincere in their interactions. Some participants mentioned that the use of cartoon characters or animation-style graphics may be better suited for younger adolescents rather than older teenagers. Fifteen (22%) participants noted a preference for animal characters, while 4 (6%) participants preferred human characters. Participants appreciated that characters were gender-inclusive with neutral names and presentation. According to parents, animal characters contributed to this inclusivity.

And so, I think that it was, it was a good, you know, they had the things that the kids could find in common with them, you know. Common things in terms of like, for instance, being anxious about things at school, you know, and things like that, it was, it was a good thing. I liked the characters.

Parent 150

#### Graphics

Parents stated that the graphics were cute and interesting but may have been designed for younger children. Only 4 (6%) participants stated that the game graphics were outdated. Participants also stated that the graphics may be basic for adolescents who often play games with advanced graphics.

In comparison to games and apps that kids use right now. They're pretty, it was pretty basic. And the, the animals were- if you're looking for teenagers or adolescents- a bit juvenile.

Parent 165

#### Navigation

Some parents (n=38, 57%), had difficulties with navigation, especially in level 1. Eleven (16%) participants sought more in-game directions for navigation while playing and suggested that keyboard controls (W, A, S, D keys) were difficult to use. For most parents, the game navigation became easier as the participants continued to play the game. Some participants (n=10, 15%), suggested gameplay may be more intuitive for a child rather than a parent.

Like, I wasn't sure I was supposed to walk around and look for things, or if it was going to read me something, or if I was just waiting for things. So, I think at least at a time in the beginning, like, figuring that part out. And then when it had me redo a level a couple of times because I did something wrong. So, I knew I was looking for a key in the beginning, but I couldn't walk around the room and look for it.

Parent 183

It was a little hard to know how to use it at the very beginning because there weren't a lot of instructions, I wasn't sure exactly what I was supposed to do when it started. But I caught on after the end of the, by the end of the 1st scene.

Parent 118

#### Pace

Participants mentioned that the game had a slow pace with opportunities to repeat the game scene when the wrong action was selected. Time burden was reported due to the amount of dialogue the participant had to read throughout the game.

There was just too much to read. Instead of like, little blurbs, it was like, big passages to read. I mean, as far as getting people's attention, unfortunately, I felt like it was more like, I was reading it, but if I wasn't doing a survey for it, I’d just be clicking, clicking, like, waiting for it to ask me a question. That way I can answer it and move on to the next subject.

Parent 155

### Theme 3: Educational Purpose of Game

#### Overview

Many participants expressed positive opinions about using a video game to educate their children on opioid medication safety. The parents reported that game-based learning would help their children remain engaged with the content. They also noted that the game would be a beneficial way for children to share what they learned with friends.

#### Perceived Main Idea of Game

When asked what they perceived to be the main educational point of the game, 23 (34%) participants reported the promotion of safe use of opioids and 13 (19%) stated prescription medications in general. Additional perceived goals of the game included education on proper opioid medication storage, which was reported by 9 (13%) participants while 22 (33%) participants reported that the overall goal of the game was medication storage in general. Encouragement to avoid sharing prescription medications was cited by 33 (49%) participants, and discussion of proper medication disposal techniques for opioids was suggested by 10 (15%) participants or medications overall was stated by 10 (15%). A portion of 17 (25%) participants further stated that the game accomplished the goals that they perceived.

The main goal of the game was to me, it was educating the public and, and people about, uh, using prescription medicine faithfully. And to me, the, the game did meet that goal. Especially like in that first part getting it, you know, securing it, locking it up. And not taking things that are, that do not have your name on it, that are not for, you. Um, and not offering it to people because you think it may help them. I think the game did- for what I played of the game- I think that did drive that point home.

Parent 147

#### Reading Level

Parents believed that the game’s reading level was more appropriate for older adolescents than elementary-aged children.

And so, it seemed like it was designed for much younger than the characters in it, which was fine, but some of the things they were saying, I don't think the younger kids would be able to read.

Parent 131

#### Target Audience

Participants reported that children of elementary or middle school age would be the primary target audience. Participants thought children 5 to 15 years of age would benefit most from the game but mentioned that the educational points are important for all ages. Dialogue and game scenes were perceived as more appropriate for older teenagers than younger children. Parents stated that their older teens may think the game less engaging due to its slow pace. However, parents reported that their children would learn from the game and gain confidence in safe prescription opioid behaviors.

I think that it can be valuable and not just the game itself, but […] perhaps the kid and parents could play it together, or the parent could watch the child playing it and it could help fill in any missing pieces or answer questions that the kid might have. I think it's a nice way to introduce the topic for a family discussion in a way that could be more engaging and that could hold the kid's interest a bit more than just straight laying it out there.

Parent 137

I definitely would recommend it to others because it obviously is extremely important and needs to be, it needs to be brought up to anyone who will listen. But I would recommend it to a younger crowd, I think.

Parent 143

#### Participant Learning Experiences

Some (n=27, 40%), participants reported that the game confirmed their existing knowledge, while 22 (33%) others reported that the game did not provide novel educational points. Two frequently reported educational outcomes included medication storage recommendations and awareness of medication disposal options. Specifically, participants realized that they do not typically store medications in a locked area and do not have a locked area available in their homes. A substantial number of participants (n=15, 22%), were not aware of available pharmacy medication disposal drop boxes until playing the game. The game also emphasized the importance of parent-to-child medication safety education wherein participants discussed improved awareness of medication safety scenarios, such as when friends of their children visit the home. Parents were also motivated to increase communication about medication safety with their children after playing the game.

That opioids should be locked up. That's that got most to me because when, because my children were a little older when I was prescribe[d] codeine and, you know, you just assume they're not going to touch it. And the childproof caps, I didn't lock mine up. But now seeing that I probably should have, because you don't know who else is in your house and can get to your medication.

Parent 199

I had no idea that there were drop boxes for medicine or that you were supposed to take it back to the pharmacy.

Parent 169

### Theme 4: Future Use of Game

#### Overview

Over half of participants (n=39, 58%), stated they would recommend MedSMARxT: Adventures in PharmaCity, and 17 (25%) other participants would provide a conditional recommendation. A small number of 5 (7%) participants mentioned that they would not recommend the game, 1 (1%) participant would conditionally not recommend it, 1 (1%) participant was indifferent, and 2 (3%) participants had mixed recommendations. Two (3%) participants did not provide a recommendation for or against the game.

#### Suggested Improvements

Improved navigation was cited as a future improvement 39 times. Participants recommended a tutorial, in the beginning, to explain navigation, pop-ups throughout the game to encourage exploring the scene, or adding a hint button. They also endorsed the use of keyboard arrow keys, a computer mouse, or touchscreens over current W, A, S, D keyboard controls.

I would have benefited from like, a little simple, like, practice round at the beginning, just a simple like, this is, I mean, this is how you move and how you jump and how you do these things.

Parent 183

To increase the pace of the game, participants desired an option to skip over dialogue especially if repeating a game scene. Participants would prefer less text dialogue and more direct questions to prompt user action. Participants recommended the incorporation of social media, live video clips, and customizable characters to increase engagement for older teens.

Participants sought additional educational content, including a scene in the beginning of the game where the main character discusses information and instructions about their medication with a physician or a parent. Participants suggested this scene could include exposure to the medication package insert, a display of warning labels found on the medication bottle, or background information on the character’s injury. More education regarding the recognition and treatment of opioid overdose was requested. Participants emphasized the importance of all users being able to see the consequences of wrong actions, even if they choose the correct actions in the game.

I would say to ensure that all the scenarios, so, even if you didn't, you know, end up in a particular sort of side quest you still got that information. Um, so maybe creating additional scenarios that so you didn't miss certain information if you answered in a particular way, and you just didn't end up on that diversion.

Parent 142

#### Applications

Specific settings thought to be right for future use include health care settings (physician offices, pharmacies, and dental offices), particularly in waiting rooms and school settings (health classes, drug awareness programs, and use with sports injuries). Participants stated that the game would be effective as a kiosk within a pharmacy or as a mobile game linked via a QR code in a health care setting.

I can see this being a game for again, in the clinic, or a doctor's office, meant to be educational or in school. I'm not sure if anyone would, like, download this game to play it just to just to play a game about learning about opioids or, you know, how to use them correctly and stuff like that, but I could definitely see this being a way to educate kids where it doesn't seem like it's a chore for them where they can use it to have fun and play a game and learn something at the same time.

Parent 167

## Discussion

### Principal Findings

Parental feedback demonstrates the acceptability and appropriateness of MedSMARxT: Adventures in PharmaCity as evaluated through parent perceptions of game design, navigation, educational purpose, and future game applications. The study findings indicate that most parents support the use of educational games to teach adolescents about opioid medication safety, emphasizing that this approach is more appealing and engaging for youths as compared to traditional learning methods. Moreover, many parents stated that they would recommend the game to others, showing its appropriateness in engaging parents and garnering their support in using the game to educate their children. Despite being a serious game directed toward use by youths, MedSMARxT: Adventures in PharmaCity also taught parents of adolescents important opioid medication safety practices, including safe storage and disposal.

### Comparison to Prior Work

Video games, specifically serious games, demonstrate the potential to improve key behavioral intentions and knowledge related to positive outcomes in various health conditions [31-33]. MedSMARxT is the first serious game to address opioid safety in adolescents [14]. As such, it is critical that the game be evaluated with relevant stakeholders to ensure key quality criteria [34-36]. Prior research has elucidated some key quality criteria in the development of serious games including the salience of the overarching goal of the game, appropriateness of the game content, enjoyment, and acceptable media presentation [37]. In order to best tailor the game to the target players, the study team has rigorously evaluated the game among groups of stakeholders. For example, the study team has conducted research with pharmacists to validate the educational content and their perceptions of using the game in pharmacies when opioid medications are dispensed. Pharmacists reported that the information presented in the MedSMARxT is accurate and age-appropriate while potentially useful for populations aside from adolescents such as parents or older adults [38].

Previous work by the study team has focused on the development of MedSMARxT wherein community-based participatory research was used to elucidate adolescent knowledge gaps and preferences for education on medication safety [39]. Adolescents most frequently reported web searches, parents, health care providers, and web-based videos as resources for medication safety information, which highlights the integral position of parents and health care professionals such as pharmacists in ensuring appropriate and correct education for adolescents [39]. In prior studies on adolescent receptiveness to using educational games to improve medication knowledge, nearly 80% of adolescent participants indicated they would be receptive to using an educational game [40]. Concordantly, this study demonstrates that parents are also receptive to the use of an educational game to educate their adolescents on opioid safety. Moreover, parents of adolescents in this study reported experience with video games at elevated levels (84%). In conjunction with previous studies, this study indicates sufficient receptiveness toward MedSMARxT for families with adolescents.

Existing research also indicates that serious games contain realistic scenarios that engage and motivate participants more than traditional learning approaches [41]. Parents in our study offered positive feedback on the game features including the media presentation (graphics) as well as the characters and individual gameplay levels. Coupled with the correct identification of the game’s overarching goal, these findings describe the potential balance of serious and game elements in the MedSMARxT game.

Although the original, intended audience is adolescents, parents likewise reported new learnings. Parents most often reported newfound knowledge about proper disposal and storage. Research has demonstrated the importance of educating patients on safe disposal. When patients are taught the proper method of disposal, they are more likely to dispose of their medications in a safe manner [42,43]. Beyond the use of a serious game to provide education to adolescents, parental involvement in adolescent medication use practices has been a protective factor against prescription drug misuse [27]. Parents can act as useful resources for information about opioid safety and important models of proper opioid use when they themselves have the requisite education. Recognizing the vital role that the family plays in the development of medication use behaviors, the National Institutes of Health [44] recommends family-based prevention programs as an effective means to reduce rates of opioid misuse among youths. Parental exposure to topics their children are learning about can promote discussions foundational to future safe medication use behaviors. Parent responses suggest that this intervention could foster family conversations around opioid and prescription medication safety by providing parents with an educational resource for both them and their children.

This study of MedSMARxT: Adventures in PharmaCity showed a perceived educational benefit for adolescents and a reported gain in parental exposure to opioid medication safety topics. Parents were able to explore realistic scenarios that their child may experience. For example, through playing level 1, parents realized that proper opioid storage in the home is not only important for their child but also for their children’s friends who may visit the home.

### Future Directions

While overall perceptions of the MedSMARxT game were positive, findings from this study suggest further improvements including a navigation tutorial, an option to skip through dialogue when repeating scenes, and explicit hints or objectives. While iterative improvement of the game itself is vital to future uptake, implementation effectiveness studies are integral to determining the best location and means for implementing this novel educational technology. Future studies will work to evaluate the effectiveness of MedSMARxT in different health care and non-health care settings to determine fit and implementation.

### Strengths and Limitations

Strengths of this study include sampling a significant parent population from across the United States, which provided a representation of many parenting opinions and styles. Second, the use of open-ended questions during the interview provided participants ample opportunity to discuss all aspects of MedSMARxT: Adventures in PharmaCity that they perceived were important. Third, analysis was carried out by 2 research team members for triangulation (GAN and LLS), guided by the principal investigator for reflexivity (OA), and validated through peer debriefing and an audit trail to support trustworthiness in terms of credibility, transferability, dependability, and confirmability.

There are limitations to this study. The first is that of self-selection bias in this sample. While this study uses a considerably large sample size for a qualitative study, parents’ self-selected participation could be present in terms of those who favor the use of educational games being more likely to participate. Second, there are limitations surrounding demographic homogeneity in this sample. The format of study activities would exclude families without access to high-quality internet or those without a computer in the home. Therefore, our study sample, while a national sample, is homogenous in terms of socioeconomic status and education level. Hence, findings of acceptability may be less generalizable to those in more difficult socioeconomic situations and those who discontinued education after high school. Recruitment was carried out across multiple streams (Qualtrics panels, social media, listserves, snowball) across a few months in order to create a sample that was as robust as possible. The study team used practices such as investigator triangulation, peer debriefing, and reflexivity to improve the trustworthiness of the data collected.

Third, social desirability bias could play a role in the positive perceptions of the parents. It is possible that when asked about their perceptions, parents were more likely to offer a positive response than a negative response. To account for this potential, the interview guide was designed and piloted to ensure neutrality in questions and prompts. Future work could use anonymous surveys to remove the researcher from data collection and decrease social desirability. The findings of this study are trustworthy given that questions were tailored to touch on multiple parts of the intervention and questions regarding potentially threatening topics were not used.

### Conclusions

The creation and use of serious educational games to teach adolescents about opioid medication safety could be an effective approach to improving youths’ knowledge of opioid prescription safety. This study investigated parent perceptions of MedSMARxT: Adventures in PharmaCity and found that parents believe this game would be beneficial for youths. MedSMARxT: Adventures in PharmaCity could be used in various non-health care and health care settings in the future to educate adolescents and inspire meaningful parent-adolescent conversations about safe opioid use.

## Appendix

Multimedia Appendix 1 Interview guide.

## Data Availability

Data collected for this study are not publicly available as it may contain private health information. Those looking to gain access to the data should contact the corresponding author.

## References

[1] Hudgins JD, Porter JJ, Monuteaux MC, Bourgeois FT (2019). Prescription opioid use and misuse among adolescents and young adults in the United States: a national survey study. PLoS Med.

[2] (2022). U.S. overdose deaths in 2021 increased half as much as in 2020 – but are still up 15%. Centers for Disease Control and Prevention.

[3] (2022). Centers for Medicare & Medicaid Services.

[4] (2019). Youth risk behavior surveillance – United States, 2019. Centers for Disease Control and Prevention.

[5] Yaster M, McNaull PP, Davis PJ (2020). The opioid epidemic in pediatrics: a 2020 update. Curr Opin Anaesthesiol.

[6] Delamerced A, Zonfrillo MR, Monteiro K, Watson-Smith D, Wills HE (2021). Factors affecting opioid management for injured children after hospital discharge. J Pediatr Surg.

[7] Binswanger IA, Glanz JM (2015). Pharmaceutical opioids in the home and youth: implications for adult medical practice. Subst Abus.

[8] Cohrs AC, Pacula RL, Dick AW, Stein BD, Druss BG, Leslie DL (2021). Trends in personal and family member opioid prescriptions prior to a diagnosis of an opioid-related problem among adolescents and young adults. Subst Abus.

[9] Voepel-Lewis T, Boyd CJ, Tait AR, McCabe SE, Zikmund-Fisher BJ (2022). A risk education program decreases leftover prescription opioid retention: an RCT. Am J Prev Med.

[10] Garbutt JM, Kulka K, Dodd S, Sterkel R, Plax K (2019). Opioids in adolescents' homes: prevalence, caregiver attitudes, and risk reduction opportunities. Acad Pediatr.

[11] Voepel-Lewis T, Farley FA, Grant J, Tait AR, Boyd CJ, McCabe SE, Weber M, Harbagh CM, Zikmund-Fisher BJ (2020). Behavioral intervention and disposal of leftover opioids: a randomized trial. Pediatrics.

[12] Abraham O, Szela L, Thakur T, Brasel K, Brown R (2021). Adolescents' perspectives on prescription opioid misuse and medication safety. J Pediatr Pharmacol Ther.

[13] Nairn SA, Audet M, Stewart SH, Hawke LD, Isaacs JY, Henderson J, Saah R, Knight R, Fast D, Khan F, Lam A, Conrod P (2022). Interventions to reduce opioid use in youth at-risk and in treatment for substance use disorders: a scoping review. Can J Psychiatry.

[14] Abraham O, Thakur T, Brown R (2019). Prescription opioid misuse and the need to promote medication safety among adolescents. Res Social Adm Pharm.

[15] Abraham O, Szela L, Rosenberger C, Birstler J, Li J, Hetzel S (2023). Examining the critical need for tailored adolescent opioid education: a national study. J Pediatr Pharmacol Ther.

[16] Abraham O, Szela L, Norton D, Stafford H, Hoernke M, Brown R (2020). Adolescents' awareness about prescription opioid misuse and preferences for educational interventions. J Am Pharm Assoc (2003).

[17] Moore SK, Grabinski M, Bessen S, Borodovsky JT, Marsch LA (2019). Web-based prescription opioid abuse prevention for adolescents: program development and formative evaluation. JMIR Form Res.

[18] Rodriguez DM, Teesson M, Newton NC (2014). A systematic review of computerised serious educational games about alcohol and other drugs for adolescents. Drug Alcohol Rev.

[19] DeSmet A, Van Ryckeghem D, Compernolle S, Baranowski T, Thompson D, Crombez G, Poels K, Van Lippevelde W, Bastiaensens S, Van Cleemput K, Vandebosch H, De Bourdeaudhuij I (2014). A meta-analysis of serious digital games for healthy lifestyle promotion. Prev Med.

[20] Baranowski T, Blumberg F, Buday R, DeSmet A, Fiellin LE, Green CS, Kato PM, Lu AS, Maloney AE, Mellecker R, Morrill BA, Peng W, Shegog R, Simons M, Staiano AE, Thompson D, Young K (2016). Games for health for children-current status and needed research. Games Health J.

[21] Egashira M, Son D, Ema A (2022). Serious game for change in behavioral intention toward lifestyle-related diseases: experimental study with structural equation modeling using the theory of planned behavior. JMIR Serious Games.

[22] Smillov M, Smith LN, Caballero NS, Cintron C, Toklu HZ (2019). Impact of an educational debate on the knowledge of college students on opioids, and factors affecting their perception about addiction. J Res Pharm.

[23] Miech R, Johnston L, O'Malley PM, Keyes KM, Heard K (2015). Prescription opioids in adolescence and future opioid misuse. Pediatrics.

[24] Susi T, Johannesson M, Backlund P (2007). Serious games: an overview. Digitala Vetenskapliga Arkivet.

[25] Abraham O, Thakur T, Brown R (2020). Developing a theory-driven serious game to promote prescription opioid safety among adolescents: mixed methods study. JMIR Serious Games.

[26] Abraham O, Rosenberger C, Tierney K, Birstler J (2021). Investigating the use of a serious game to improve opioid safety awareness among adolescents: quantitative study. JMIR Serious Games.

[27] Nargiso JE, Ballard EL, Skeer MR (2015). A systematic review of risk and protective factors associated with nonmedical use of prescription drugs among youth in the United States: a social ecological perspective. J Stud Alcohol Drugs.

[28] (2020). NVivo qualitative data analysis. Lumivero.

[29] Braun V, Clarke V (2006). Using thematic analysis in psychology. Qual Res Psychol.

[30] (2020). Microsoft Excel. Microsoft.

[31] Baranowski T, Baranowski J, Thompson D, Buday R, Jago R, Griffith MJ, Islam N, Nguyen N, Watson KB (2011). Video game play, child diet, and physical activity behavior change a randomized clinical trial. Am J Prev Med.

[32] Beale IL, Kato PM, Marin-Bowling VM, Guthrie N, Cole SW (2007). Improvement in cancer-related knowledge following use of a psychoeducational video game for adolescents and young adults with cancer. J Adolesc Health.

[33] Lieberman DA (2001). Management of chronic pediatric diseases with interactive health games: theory and research findings. J Ambul Care Manage.

[34] Jacquez F, Vaughn LM, Wagner E (2013). Youth as partners, participants or passive recipients: a review of Children and Adolescents in Community-Based Participatory Research (CBPR). Am J Community Psychol.

[35] Israel BA, Schulz AJ, Parker EA, Becker AB, Community-Campus Partnerships for Health (2001). Community-based participatory research: policy recommendations for promoting a partnership approach in health research. Educ Health (Abingdon).

[36] Fleming TM, Bavin L, Stasiak K, Hermansson-Webb E, Merry SN, Cheek C, Lucassen M, Lau HM, Pollmuller B, Hetrick S (2017). Serious games and gamification for mental health: current status and promising directions. Front Psychiatry.

[37] Caserman P, Hoffmann K, Müller P, Schaub M, Straßburg K, Wiemeyer J, Bruder R, Göbel S (2020). Quality criteria for serious games: serious part, game part, and balance. JMIR Serious Games.

[38] Abraham O, Slonac E, Paulsen Z (2023). Pharmacists' perspectives on MedSMARxT: a serious game to educate youth about opioid safety. J Am Pharm Assoc (2003).

[39] Abraham O, Szela L, Brasel K, Hoernke M (2022). Engaging youth in the design of prescription opioid safety education for schools. J Am Pharm Assoc (2003).

[40] Abraham O, Rosenberger CA, Birstler J, Tierney K (2022). Examining adolescents' opioid knowledge and likelihood to utilize an educational game to promote medication safety. Res Social Adm Pharm.

[41] Boyle E, Connolly TM, Hainey T (2011). The role of psychology in understanding the impact of computer games. Entertain Comput.

[42] Harbaugh CM, Malani P, Solway E, Kirch M, Singer D, Englesbe MJ, Brummett CM, Waljee JF (2020). Self-reported disposal of leftover opioids among US adults 50-80. Reg Anesth Pain Med.

[43] Schäfer WLA, Johnson JK, Wafford QE, Plummer SG, Stulberg JJ (2021). Primary prevention of prescription opioid diversion: a systematic review of medication disposal interventions. Am J Drug Alcohol Abuse.

[44] (2003). Preventing drug abuse among children and adolescents: a research-based guide for parents, educators, and community leaders. National Institute on Drug Abuse.

