# Supplementary material for: Parents’ Perceptions of a Serious Game for Educating Families on Prescription Opioid Safety: Qualitative Pilot Study of MedSMARxT: Adventures in PharmaCity
Source: JMIR Serious Games. 2023 Sep 12;11:e49382. doi: 10.2196/49382 (PMC10523225; doi:10.2196/49382)
Supplement: Multimedia Appendix 1 [file games_v11i1e49382_app1.docx]

**The first questions are about the game and elements of the game.**

What did you think of the video game?

What did you like or dislike about the game, and why?

What changes could be made to improve the game?

Tell me how you feel about the characters in the game. (Prompt, if needed: Were they realistic? What did you like/dislike about them? How could we improve them?)

How did you feel about the scenarios presented in the game? (Prompt, if needed: Were they realistic? Engaging?)

Would you recommend this game to others, why/why not?

**The following questions are about what you learned from the game.**

What do you feel was the main goal of the game? How could the game be improved in order to meet this goal?

What did you learn from this game, if anything?

**Now, I’d like to ask you some questions about your experience with games.**

Do you have any experience with playing video games? Please tell me about your experience with video games.

(If they have little to no experience): Is there a reason you don’t play video games? If yes, why not?

(If they have little to no experience): Is there anything that could be changed that would make you want to play them?

(If they do play games): What type of video games do you prefer?

What games are you currently playing? Why do you enjoy playing these games?

Do you play games on your phone / board games? If so, what games? Why do you play them?

How do you feel about playing games that are educational?

Have you played any other educational games? If so, what do you like or dislike about these games?

Is there anything else you’d like to add?
